# Immune Profile of Exosomes in African American Breast Cancer Patients Is Mediated by Kaiso/THBS1/CD47 Signaling

**DOI:** 10.3390/cancers15082282

**Published:** 2023-04-13

**Authors:** Md Shakir Uddin Ahmed, Brittany D. Lord, Benjamin Adu Addai, Sandeep K. Singhal, Kevin Gardner, Ahmad Bin Salam, Anghesom Ghebremedhin, Jason White, Iqbal Mahmud, Rachel Martini, Deepa Bedi, Huixian Lin, Jacqueline D. Jones, Balasubramanyanam Karanam, Windy Dean-Colomb, William Grizzle, Honghe Wang, Melissa Davis, Clayton C. Yates

**Affiliations:** 1 Department of Biology and Center for Cancer Research, Tuskegee University, Tuskegee, AL 36088, USA; 2 Bangladesh Council of Scientific and Industrial Research, Dhaka 1205, Bangladesh; 3 Department of Genetics, University of Georgia, Athens, GA 30602, USA; 4 School of Veterinary Medicine, Tuskegee University, Tuskegee, AL 36088, USA; 5 Department of Pathology, School of Medicine and Health Sciences, University of North Dakota, Grand Forks, ND 58202, USA; 6 Department of Biomedical Engineering, School of Electrical Engineering and Computer Science, University of North Dakota, Grand Forks, ND 58202, USA; 7 Department of Pathology and Cell Biology, Columbia University Medical Center, New York, NY 10032, USA; 8 Department of Pathology, Immunology and Laboratory Medicine, College of Medicine, University of Florida, Gainesville, FL 32610, USA; 9 Department of Surgery, Weill Cornell Medicine, New York, NY 10065, USA; 10 Department of Biological and Environmental Sciences, Troy University, Troy, AL 36082, USA; 11 Piedmont Oncology-Newnan, Newnan, GA 30265, USA; 12 Department of Pathology, School of Medicine, The University of Alabama at Birmingham, Birmingham, AL 35233, USA; 13 Department of Pathology, Johns Hopkins University School of Medicine, Baltimore, MD 21287, USA; 14 Sidney Kimmel Comprehensive Cancer Center, Johns Hopkins University School of Medicine, Baltimore, MD 21287, USA

**Keywords:** Kaiso, breast cancer, exosomes, immune signaling, cancer health disparity, tumor microenvironment, inflammation, African Americans, CD47, THBS1, SIRPA

## Abstract

**Simple Summary:**

African American (AA) women with breast cancer are more likely to have higher inflammation and a stronger overall immune response than European American (EA) women. In this report, we demonstrate the biological role of Kaiso in the immune signaling of breast cancer exosomes. Our findings indicate that Kaiso directly represses tumor suppressor THBS1, and this is associated with increased expression of CD47 and signal regulatory protein (SIRPA). Kaiso depletion attenuated tumor formation in athymic nude mice, and this is associated with increased infiltration of M1 macrophages. In vitro studies using THP1 cells that were treated with exosomes from Kaiso-depleted cells polarize towards the M1 phenotype with increased phagocytosis of cancer cells compared to high Kaiso control exosome, which polarizes towards an M2 phenotype. In summary, these findings reveal that Kaiso modulates the macrophage-mediated immune escape of cancer cells through exosome signaling, which may be related to poorer outcomes, especially for AA women.

**Abstract:**

African American (AA) women with breast cancer are more likely to have higher inflammation and a stronger overall immune response, which correlate with poorer outcomes. In this report, we applied the nanostring immune panel to identify differences in inflammatory and immune gene expression by race. We observed a higher expression of multiple cytokines in AA patients compared to EA patients, with high expression of CD47, TGFB1, and NFKB1 associated with the transcriptional repressor Kaiso. To investigate the mechanism associated with this expression pattern, we observed that Kaiso depletion results in decreased expression of CD47, and its ligand SIRPA. Furthermore, Kaiso appears to directly bind to the methylated sequences of the THBS1 promotor and repress gene expression. Similarly, Kaiso depletion attenuated tumor formation in athymic nude mice, and these Kaiso-depleted xenograft tissues showed significantly higher phagocytosis and increased infiltration of M1 macrophages. In vitro validation using MCF7 and THP1 macrophages treated with Kaiso-depleted exosomes showed a reduced expression of immune-related markers (CD47 and SIRPA) and macrophage polarization towards the M1 phenotype compared to MCF7 cells treated with exosomes isolated from high-Kaiso cells. Lastly, analysis of TCGA breast cancer patient data demonstrates that this gene signature is most prominent in the basal-like subtype, which is more frequently observed in AA breast cancer patients.

## 1. Introduction

In the United States, breast cancer is the second most common cause of cancer deaths among women, surpassed only by lung cancer, affecting African American (AA) women at a 40% higher rate compared to European American (EA) women [1]. The incidence of breast cancer for AA women is equal to that of EA women; however, AA women experience early onset of this disease and significantly higher mortality rates. Breast cancers are typically characterized based on the expression of three tumor markers, namely estrogen receptor (ER), progesterone receptor (PR), and human epidermal growth factor receptor (HER2) [2]. The racial disparity in cancer is multi-factorial; however, African American women display some of the characteristic features of triple negative breast cancer (TNBC), which lack receptor expression and are associated with the worse outcome [2,3].

Recently, the breast cancer incidence and mortality disparity observed within the AA population has been linked to innate immune-related genes, inflammation, lymphocyte infiltration, and distribution of immune genes [4,5,6,7,8]. Compared to EA breast cancer patients, AA breast cancer patients have higher levels of inflammatory biomarkers, cytokines, and CD8^+^ T cell density [6,9,10,11,12]. Breast tumors from AA patients show higher interferon signatures, macrophages, and immune dysfunction scores [13,14] as well as higher fractions of leukocytes and lymphocytes [15,16,17]. Furthermore, in the TCGA breast cohort, AA tumors show significantly upregulated expression of immune checkpoint inhibitors, such as PD-1, PD-L1, and CTLA-4 [18]. In comparison to EA tumors, AA women have higher expressions of basal-like 1, basal-like 2, and immune-modulatory signatures [18]. Both AA breast and prostate cancer patients demonstrate changes in gene expression signatures relative to protective innate immune variants [19,20] as well as the epithelial to mesenchymal transition (EMT) and elevated immune-related pathways in comparison to EA patients [21]. For example, our lab and others have demonstrated that Kaiso, a POZ transcriptional repressor, is elevated in AA and native African women [22,23], and deletion of Kaiso expression affects numerous pathways, including immune regulation [24].

The capacity of cancer cells to escape immune surveillance is a hallmark of cancer progression and metastasis [25]. Several immune cells and cell surface molecules regulate the interaction between immune escape and breast cancer outcomes. To communicate in the tumor microenvironment as well as with distal parts of the body, cancer cells utilize extracellular vesicles (EV). A major component of EVs are exosomes, which are nano-sized extracellular vesicles, containing various macromolecules, including genetic materials, proteins, and lipids, which can be transferred to recipient cells from donor cells [26,27,28,29,30]. Exosomes inherit different capabilities from the parental cells, thereby functioning in the mediation of cell–cell communication in tumor cells to promote EMT, immune escape, and metastasis [31,32]. Crosstalk between cancer cells and immune cells is mediated by exosomes [33,34,35,36,37]. Further, several immune markers associated with a poor prognosis are highly expressed in cancer cells and are present in exosomes [34,36,38,39]. However, the mechanisms underlying the cell–cell communication by exosomes modulating the immune signaling in breast cancer with poor prognosis have not been explored thoroughly, particularly for AA breast cancer patients. In this study, we report that the THSP1-CD47 signaling axis is regulated by Kaiso, which modulates immune profiling, especially in AA breast cancer exosomes. 

Herein we demonstrate that AA breast cancer exosomes demonstrate increased expression of multiple inflammatory cytokines. To identify the mechanism associated with this increased inflammation, we observed that tumor-promoting genes such as CD47, TGFB1, and NKFB1 are linked with Kaiso expression in AA patients. Furthermore, depletion of Kaiso in MDA-MB-231 cells demonstrates a lower expression of CD47 and its ligand SIRPA, but higher expression of THBS1. We further demonstrate that Kaiso directly binds to methylated regions of the THBS1 promoter, resulting in the silencing of THBS1 expression. In vivo Kaiso depleted MDA-MB-231 cells (sh-Kaiso cells) exhibited decreased tumor formation and metastasis capacity and were associated with increased expression of the M1 macrophage marker CD86 as well as increased phagocytosis. In addition, MCF7 cells treated with Kaiso-depleted exosomes show a lower expression of Kaiso, CD47, and SIRPA, and a higher expression of THBS1. Similarly, THP1 macrophages treated with Kaiso-depleted exosomes highly express CD86, compared to high expression of the M2 macrophage marker CD206 in cells treated with Kaiso control exosomes. Lastly, analysis of TCGA breast cancer patient data demonstrates that this gene signature is most prominent in the basal-like subtype, which is more frequently observed in AA breast cancer patients. Thus, these findings suggest that Kaiso is a regulator of the THBS1/CD47/SIRPA signaling axis, thereby modulating immune signaling by exosomes to evade immune surveillance, particularly in AA breast cancers.

## 2. Materials and Methods

### 2.1. Study Population and Analysis

From 30 breast cancer patients (14 AA and 16 EA), blood samples were collected following IRB approval from the University of Alabama at Birmingham and the University of Georgia. Descriptions of the patients are in Supplementary Table S1. Two independent dataset with BRCA cell lines, CCLE breast cancer cell lines (n = 69) [40], and BRCA cell lines (n = 51) were analyzed as validation sets [41]. mRNA data were downloaded from the TCGA Research Network https://www.cancer.gov/about-nci/organization/ccg/research/structural-genomics/tcga (accessed on 3 March 2019). Among 1098 samples, we used a total of 153 AA or black patients and 645 EA or white patients to observe gene expression values representing differential gene expression. Genomic data were processed using JMP and R software. TCGA mRNA and protein (CPTAC) data were also analyzed and visualized using the Xena platform [42] and ULCAN analysis platform [43].

### 2.2. Cell Culture

The breast cancer cell line MDA-MB-231 and MCF7 cells, purchased from ATCC, were maintained and grown in Dulbecco’s modified Eagle’s high glucose (4.5 g/L) medium. This medium was supplemented with fetal bovine serum (FBS; 10%), penicillin-streptomycin (10,000 IU/mL and 10,000 μg/mL), sodium pyruvate (100 mM), non-essential amino acids (1×), and L-glutamine (200 mM). All cells were grown at 37 °C with 90% humidity, 5% CO_2_, and 95% air. Kaiso-depleted MDA-MB-231 cells, named sh-Kaiso cells, and Kaiso-scrambled MDA-MB-231 cells, named sh-Scr, were grown in DMEM media using the supplements mentioned here, except that puromycin (0.8 µg/mL) was added in the media to maintain selection. THP1 cells, also purchased from ATCC, were maintained and grown in RPMI-1640 medium with the above-mentioned supplements.

### 2.3. Antibodies

Anti-Kaiso antibody (ab12723), anti-CD47 antibody (ab175388), anti-THBS1 antibody (ab86762), anti-SIRPA antibody (ab53721), anti-TGFB1 antibody (ab92486), and anti-β-actin antibody (ab8227) were purchased from Abcam, Fremont, CA, USA. All primary antibodies for immunohistochemistry (IHC) and immunoblots were used at 1:100 to 1:1000 dilutions based on the manufacturer’s recommendation. Secondary antibodies, including goat anti-mouse IgG H&L (HRP) antibody (ab6789) and goat anti-rabbit IgG H&L (HRP) antibody (ab6721), were purchased from Abcam, Fremont, CA, USA. For immunofluorescence assays, anti-CD86 antibody (ab64693) and anti-CD206 antibody (ab64693) were used at a dilution of 1:100. Secondary antibodies, including anti-mouse antibody (ab86762) and anti-rabbit antibody (ab86762), were purchased from Abcam, Fremont, CA, USA.

### 2.4. Generation of Stable Kaiso-Depleted MDA-MB-231 Cells

Kaiso depletion of MDA-MB-231 cells was performed as previously described [44] Briefly, 6 μg of sh-Kaiso vector (pRS-sh-Kaiso plasmid) or control vector (pRS-Kaiso scrambled plasmid), were utilized to transfect MDA-MB-231 cells using the Turbofect transfection reagent (Thermo Scientific, Waltham, MA, USA), according to the manufacturer’s instructions. After 48 h of post-transfection, cells were treated with 0.8 μg/mL of puromycin (Invitrogen, Carlsbad, CA, USA) to select cells with stable Kaiso knockdown. Kaiso depletion was confirmed using Western blot analysis from individual transfected clones and normal MDA-MB-231 cells. Clones exhibiting the most significant Kaiso knockdown were selected for further studies [44].

### 2.5. Exosome Isolation and Characterization

Briefly, all breast cell lines were cultured for 48 h in FBS-depleted media and xosomes were isolated according to established protocols [45]. Sequential centrifugations were used to discard dead cells and cellular debris. The supernatant was then filtered through 0.2-μm filters [46,47]. Exosomes were isolated using Exoeasy maxi kits (Qiagen, Germantown, MD, USA) according to the manufacturer’s protocol. Briefly, 1 volume of binding buffer was added to 1 volume of filtered sample, then the sample mixture was transferred to the spin column followed by centrifugation at 500× *g* for 1 min. After discarding the flow-through, 10 mL of wash buffer was added to the sample and centrifuged at 5000× *g* for 1 min. Next, 400 µL elution buffer was added to the membrane, and 1 min incubation was followed by centrifuging at 5000× *g* for 5 min to collect the extracted exosomes. Exosome proteins were extracted by the use of RIPA lysis buffer and extraction buffer (ThermoFisher Scientific, Waltham, MA, USA), according to manufacturer’s manuals. Protease Inhibitor Cocktail (ThermoFisher Scientific, Waltham, MA, USA) was added during protein extraction. Exosome protein markers were quantified using Pierce™ BCA Protein Assay kits (ThermoScientific, Waltham, MA, USA), according to the manufacturer’s protocol. The absorbance was read at 562 nm on a microplate reader (BioTek, Winooski, VT, USA). The expressions of exosome makers were confirmed using Exo-Check Exosome Antibody Array (System Biosciences, Palo Alto, CA, USA) kits, as recommended by the manufacturer. The membranes were developed with Super Signal West Femto Maximum Sensitivity Substrate (ThermoFisher Scientific, Waltham, MA, USA) and analyzed using ChemiDoc. Exosome sizes were characterized using nanoparticle tracking analysis in a Nanosight NS300 (Malvern Panalytical, Malvern, UK). Briefly, the samples were injected and captured in 5 videos for 30 s, then the average was used to assess the size distribution of exosomes.

Serum-derived patient exosomes from 14 AA and 16 EA breast cancer patients were extracted from whole blood using the exoEasy Maxi Kit (Qiagen, Germantown, MD, USA) following the manufacturer’s protocol. In brief, 0.3–0.4 mL of serum was filtered using a 0.22 μm syringe filter to exclude larger particles. Samples were run through the exoEasy spin column as described above using the appropriate buffers. Following centrifugation, samples were eluted in PBS by centrifuging at 5000× *g* for 5 min and stored at −20 °C until downstream analysis.

### 2.6. RNA Extraction and Quantitative Real-Time PCR

TRIzol Reagent (ThermoFisher Scientific, Waltham, MA, USA) was used to extract RNA according to the manufacturer’s instructions. The reagent was added to cells following experimental conditions. RNA was further purified using 100% ethanol, then the concentration of RNA was quantified with a Nanodrop 2000 (ThermoFisher Scientific, Waltham, MA, USA). RNA reverse transcription to synthesize cDNA was performed using SuperScript II Reverse Transcriptase Kits (Life Technologies, Carlsbad, CA, USA) according to the manufacturer’s instructions. Quantitative real-time PCR was performed using SYBR Green kits (ThermoFisher Scientific, Waltham, MA, USA), and primer sets for Kaiso, CD47, SIRPA, THBS1, and TGFB1 (Supplementary Table S2) were designed using Primer 3 (http://bioinfo.ut.ee/primer3-0.4.0/, accessed on 10 January 2018) [48]. Results were calculated using the ΔCt method [49], and the gene expression was normalized to the β-actin expression [50,51,52].

### 2.7. NanoString Human PanCancer Immune Profiling Panel Analysis

Exosomes extracted from the serum of AA and EA breast cancer patients, as well as exosomes extracted from sh-Kaiso and sh-Scr cells (5 × 10^6^), were used for extracting RNA using Qiagen RNeasy kits (Qiagen, Germantown, MD, USA). Extracted RNA was analyzed for 730 immune-related genes by utilizing the nCounter Human PanCancer Immune profiling panel according to the manufacturer’s instruction (Nanostring Technologies, Seattle, WA, USA). In brief, 100 ng of RNA was hybridized with sequence-specific barcoded reporter probes at 65 °C for 24 h. After hybridization, the samples were placed into a NanoString Prep Station in which the target-probe complex was immobilized and aligned on the nCounter cartridge. Sample cartridges were read by a nCounter Digital Analyzer for counting barcodes specific to each target, and the corresponding data were collected using the Nanostring Sprint instrument. nSolver 3.0 analysis software was used for analyzing the data [53].

### 2.8. Treatment with 5-aza-2-Deoxycytidine (5-aza)

MDA-MB-231 cells were incubated to around 60% confluent cells and then treated with 5 μM of 5-aza for 72 h. The 5-aza treatment in culture media was replaced every 24 h. After 72 h, cells were collected, and cell lysates were prepared for chromatin immunoprecipitation assays (ChIP) or q-PCR analysis.

### 2.9. Methylation-Specific PCR

The CpG islands associated with the THBS1 promoter were predicted using the CpG prediction algorithm (http://www.urogene.org/cgi-bin/methprimer/methprimer.cgi, accessed on 10 January 2018) [54]. Subsequently, primers for methylation-specific PCR (Supplementary Table S3) were designed by using the Methprimer algorithm (http://www.urogene.org/methprimer/index.html, accessed on 10 January 2018).

### 2.10. Immunoblotting

Total cell lysates were heated at 95 °C for 5 min in Laemmli sample buffer (SigmaAldrich, St. Louis, MO, USA), separated using SDS polyacrylamide gels, and then electrophoretically transferred to PVDF membranes (Millipore, Burlington, MA, USA). The membranes were incubated for 1 h in 5% nonfat milk with Tris-buffered saline (TBS) solution containing 0.1% Tween 20 (TBST). The membranes were then incubated overnight in a cold room on a shaker with the appropriate primary antibodies diluted in 5% nonfat milk with TBST. After three times washing in TBST solution, membranes were incubated with horseradish peroxidase (HRP)–conjugated anti-mouse or anti-rabbit secondary antibody for 1 h (Abcam, Fremont, CA, USA). The membranes were then washed in TBST solution, incubated for 1 min using ECL mix, and then developed using a chemiluminescence detection system.

### 2.11. ChIP Assays

ChIP assays were performed using an EZ-Magna ChIP™ G—Chromatin Immunoprecipitation Kit (Millipore Sigma, St. Louis, MO, USA) to determine Kaiso binding to the THBS1 methylated promoter. In brief, 5-aza-treated and untreated MDA-MB-231 cells were cross-linked using 1% formaldehyde at room temperature for 10 min, and a 1:10 volume of 10× glycine was added to quench the cross-linking reaction for 5 min at room temperature. After washing using ice-cold 1 × PBS, cells were pelleted and lysed using an SDS lysis buffer supplemented with a protease cocktail inhibitor. The lysates were sonicated on ice to shear to the chromatin to 200 and 1500 bp. An mount of 5 μL was saved for an input DNA control from each sonicated sample. Sheared crosslinked chromatin was incubated with protein G magnetic beads using dilution buffer containing protease inhibitor cocktail II. Kaiso antibody (Abcam, Boston, MA, USA), mouse IgG (negative control), and anti-RNA polymerase II (positive control) were used for chromatin immunoprecipitation. ChIP and input DNA (2 ng each) were then used for real-time PCR analysis. Primers were designed to amplify a 239-bp fragment of the methylated CpG islands of the THBS1 promoter region: 5′-AGTATCCACCTCTCGCCATC-3′ and 5′-GGCTTGGGAGCACTAGAACTT-3′.

### 2.12. Animal Studies

Six–eight-week-old athymic nude female mice (Envigo Inc., Indianapolis, IN, USA) were injected with sh-Scr or sh-Kaiso cells into the mammary fat pads. Tumor growth was monitored and measured using Vernier calipers, and the volume of the tumor (in mm^3^) was measured using the following formula: length/2 × width^2^, 2–3 times per week [44].

### 2.13. Histology and Immunohistochemistry (IHC)

Harvested tumor tissues were fixed in 4% formalin for 72 h and then embedded in paraffin followed by storing at room temperature until use. The paraffin-embedded tissues were sliced to have six tissue sections with a thickness of 5 μm; the sections were then mounted on individual slides. The paraffin embedded tissue slides were then processed and stained with either hematoxylin & eosin (H&E) or by using a corresponding antibody. IHC analyses of tissue sections were performed as described previously [55]. The tissue sections were incubated with primary antibody for 1 h at room temperature, using the following antibodies: anti-Kaiso 6F mouse monoclonal antibody (1:500), anti-CD47 rabbit polyclonal antibody (1:500), anti-SIRPA rabbit polyclonal antibody (1:50), or anti-THBS1 rabbit polyclonal antibody (1:1000). After washing, the sections were incubated with goat anti-mouse or donkey anti-rabbit secondary antibody (1:1000) for 40 min at room temperature. Images were then taken from the representative tissue areas of H&E and IHC-stained tissue sections using a Nikon Eclipse 50 light microscope.

### 2.14. Phagocytosis with BMDM

For fluorescence microscopic analysis of phagocytosis, BMDMs stained with CellTracker™ Green (Thermo Fisher Scientific) were seeded to 6-well-plates at a density of 2.5 × 10^5^ and the mixtures of 1 × 10^6^ of MDA-MB-231 cells stained with CellTracker™ Red (Thermo Fisher Scientific). After 4 h of co-incubation, the engulfment of cells by macrophages was analyzed as a red positive signal related to phagosome formation in macrophages by fluorescence microscopy [56].

### 2.15. Statistics

All quantitative experiments were performed in triplicate. Statistical analyses were performed using Microsoft Excel, GraphPad Prism, or JMP software, as appropriate. Paired and unpaired *t*-tests were performed for two groups and one-way ANOVA was used for more than two groups to determine standard deviation, standard error, and statistical significance between experimental and control groups. In the case of mice tumor volume, the F-test was performed. Values with *p*  ≤  0.05 were considered statistically significant.

## 3. Results

### 3.1. Characterization of Exosomes

Exosomes were extracted and purified from the culture supernatants of sh-Scr and sh-Kaiso MDA-MB-231 cell lines (Supplementary Figure S1A). Isolated exosomes expressed surface markers such as CD63, CD81, Tsg101, flotillin, and EpCam; however, they lacked GM130 expression (Supplementary Figure S1B). Nanosight analysis demonstrated that exosomes from both sh-Scr and sh-Kaiso cells exhibited size distributions within 80–220 nm in diameter (Supplementary Figure S1C), with average sizes of 195 nm to 205 nm in diameter (Supplementary Figure S1D,F), which are characteristic sizes of exosomes [31,57,58,59]. Nanosight images revealed that the isolated exosome-like particles consisted primarily of round vesicles (Supplementary Figure S1E,G).

### 3.2. Immune Function Disparity among AA and EA Breast Cancer Exosomes

**Figure 1:**
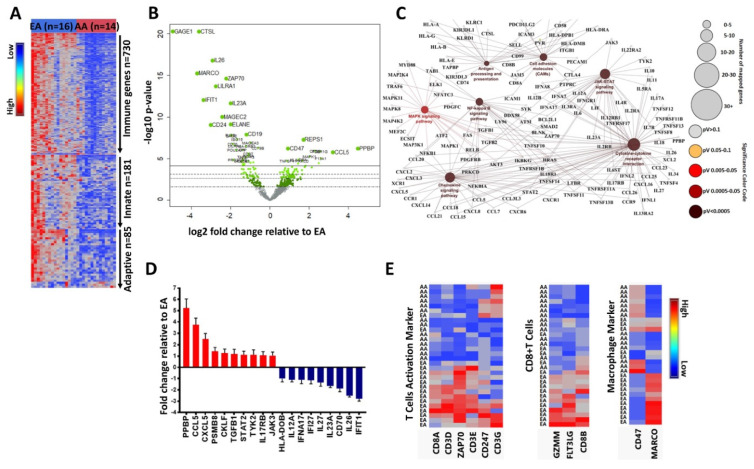
Immune function genes from AA and EA breast cancer exosomes show a racial disparity. (**A**) Nanostring PanCancer Immune gene analysis, which includes 730 immune function genes, showed that, relative to EA breast cancer exosomes, AA breast cancer exosomes had lower expression of overall immune genes as well as innate immune response genes and adaptive immune response genes. (**B**) A volcano plot showed the differential expression of immune genes in AA and EA breast cancer exosomes, where a few genes such as CD47, CCL5, PPBP, and TGFB1 were increased in AA exosomes, whereas CD24, IL26, CTSL, and GAGE1 were increased in EA exosomes. (**C**) Cytoscape analysis showed that genes interacting through the chemokine signaling pathway, the cytokine–cytokine receptor pathway, the MAPK signaling pathway, and the NF-kB signaling pathway were upregulated in AA exosomes as compared to EA exosomes (**D**) Differential expressions of chemokine, cytokine, and interleukin function genes in AA and EA breast cancer exosomes, notably PPBP, CXCL5, CCL5, and TGFB1 (involved in cancer progression) were increased in AA exosomes. (**E**) Hierarchical clustering showed that T-cell activation genes and CD8+ T cell genes were increased in EA exosomes, whereas macrophage marker genes, especially the expression CD47, were increased in AA exosomes.

Breast cancers from AA women have a high expressions of immunosuppressive markers [60], such as M2 macrophages, and decreased cytotoxic T cell activity. To understand these differences in immune gene expression, we analyzed exosomes isolated from the serum of 14 AA and 16 EA breast cancer patients using the Nanostring Pan-Cancer Immune Profiling Panel. Overall immune gene expression, including innate and adaptive immune response genes, were decreased in AA exosomes as compared to EA exosomes (Figure 1A). We also observed differential expression of genes related to cytokines, chemokines, and interleukins in AA and EA exosomes (Supplementary Figure S2), with CCL5, PPBP, and TGFB1 demonstrating the most significantly increased expression in AA exosomes (Figure 1B,D). In addition, genes interacting through the chemokine signaling pathway, the cytokine–cytokine receptor pathway, the MAPK signaling pathway, and the NF-kB signaling pathway were upregulated in AA exosomes as compared to EA exosomes (Figure 1C). Analysis of immune function cell types demonstrated that genes associated with CD8+ T cell activation were decreased in AA exosomes, (Figure 1E). CD47, which is an anti-phagocytosis marker [61,62,63] was increased in AA exosomes (Figure 1E). These data suggest that racial differences in macromolecule content in cargo-caring exosomes contribute to the immunosuppressive microenvironment for AA women with breast cancer.

### 3.3. Kaiso Represses the Expression of Several Immune Genes

**Figure 2:**
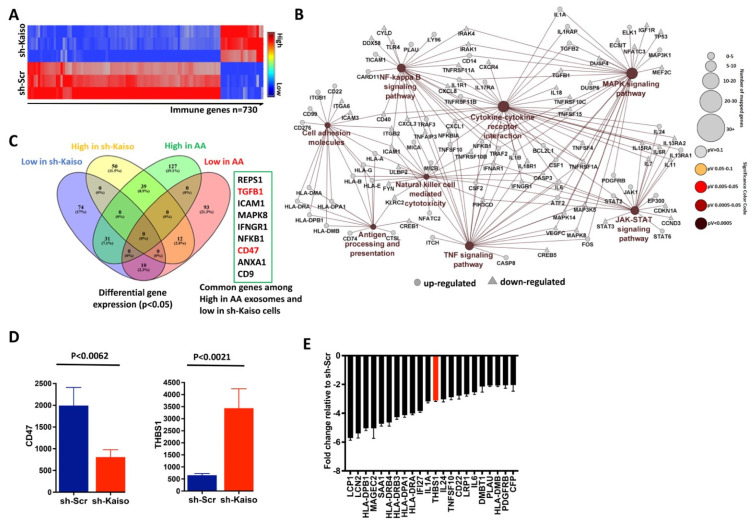
Kaiso represses the expression of several immune genes, including THBS1. (**A**). Hierarchical clustering of differentially expressed genes from PanCancer Nanostring Immune Profiling showed that there were fewer immune genes expressed in sh–Kaiso cells as compared to control sh-Scr cells. (**B**). Cytoscape analysis shows that genes interact through the chemokine signaling pathway, the cytokine–cytokine receptor pathway, the MAPK signaling pathway, and the NOD–like receptor signaling pathway, and the NF–kappa B signaling pathway was downregulated in sh-Kaiso cells as compared to sh-Scr cells. (**C**). A Venn diagram shows the association of immune genes between genes that were upregulated in AA exosomes and genes downregulated in sh-Kaiso cells (identified by color code). A total of 31 genes, including CD47 and TGFB1, were common genes among these two groups. (**D**). Kaiso-depleted cells showed significantly lower expression of CD47 with significantly higher expression of THBS1 as compared to sh–Scr cells. The statistical method used was the two–tailed paired *t* test. (**E**). Nanostring immune profiling showed that Kaiso repressed several genes, including THBS1, based on the downregulated gene expression in sh–Scr cells.

Previously, we and others have demonstrated that expression of the transcriptional repressor, Kaiso, is associated with breast cancers in women of African ancestry [22,24], and MDA-MB-231 cells with depleted Kaiso demonstrate significant differences in EMT-related genes [24]. Therefore, to determine the function of Kaiso on breast cancer immune function signaling, we utilized immune gene profiling to explore the relationship of Kaiso with differentially expressed genes from AA breast cancer patients. Kaiso-depleted cells demonstrated a decrease in the immune-related genes as compared to sh-Scr cells (Figure 2A). Cytoscape analysis showed that genes interacting through the chemokine signaling pathway, the cytokine–cytokine receptor pathway, the MAPK signaling pathway, the NOD-like receptor signaling pathway, and the NF-kB signaling pathway were downregulated in sh-Kaiso cells when compared to sh-Scr cells (Figure 2B). To depict the association of genes among the highly expressed genes (*p* ≤ 0.05) in AA exosomes and low expressed genes in sh-Kaiso cells, we analyzed the association of the genes by using a Venn diagram and found 31 correlated genes. Consistent with our findings in the differential gene expression in AA and EA exosomes, CD47 was one of the overlapping genes (Figure 2C). Because mRNA expression from exosome (Figure S3) and mRNA expression from the cell lines displaced similar expression profiles as from cells (Figure 2A), we utilized cell line gene expression to identify genes that Kaiso are directly regulated by. Kaiso depletion significantly decreased the expression of CD47 but increased THBS1 expression (Figure 2D). Then, to discover the biological function of Kaiso as a transcriptional repressor, we analyzed significantly downregulated genes in sh-Scr cells; THBS1 was downregulated (fold change = ~3.5, *p* ≤ 0.0021) (Figure 2E). Therefore, Kaiso depletion shows a significantly lower expression of CD47 (*p* ≤ 0.0062) with a significantly higher expression of THBS1 (*p* ≤ 0.0021) as compared to control cells, indicating the role of Kaiso in this THBS1/CD47 axis.

### 3.4. Kaiso Expression Inversely Correlates with THBS1 Expression

**Figure 3:**
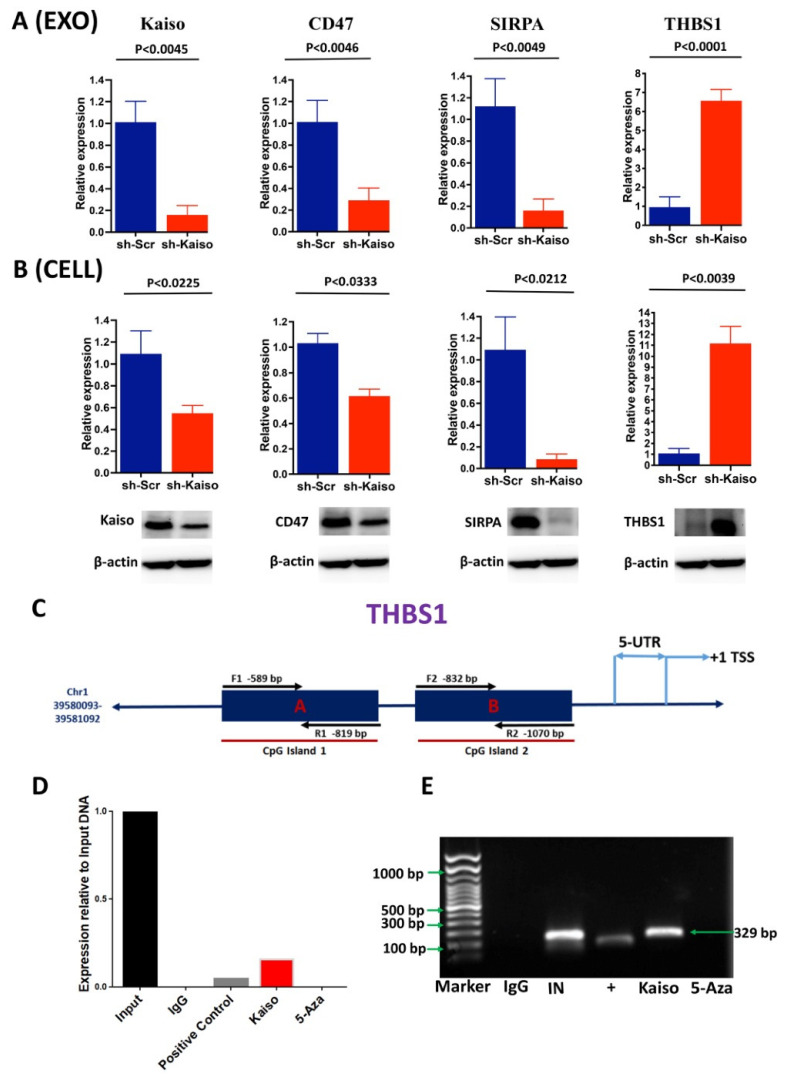
Kaiso directly regulates the expression of the tumor suppressor, THBS1. (**A**) Kaiso depletion resulted in significantly higher expression of THBS1 in exosomes from MDA-MB-231 cells, whereas the expression of CD47 and SIRPA was decreased. The statistical method used was the two-tailed paired *t* test. (**B**) As consistent with exosomes, both gene expression, and protein expression analysis revealed that Kaiso depletion resulted in a significantly high expression of THBS1 in MDA-MB-231 cells, whereas the expressions of CD47 and SIRPA were decreased. The statistical method used was the two-tailed paired *t* test. (**C**) Schematic illustration of the THBS1 promoter showing the Kaiso binding site on the CpG island. Two oligonucleotides were designed as CHIP primers from two different sites where Kaiso binds in a methylation-dependent manner. (**D**,**E**) CHIP experiments with MDA-MB-231 chromatin demonstrated that Kaiso binds to the methylated region of the THBS1 promoter and that 5-aza reversed the binding. Both PCR and gel electrophoresis confirmed the binding. Input, 1% input DNA; Positive, anti-RNA Polymerase II and Negative, normal mouse IgG; 5-aza, 5-aza-2′-deoxycytidine. The uncropped blots are shown in Supplementary File S1.

To demonstrate the biological function of Kaiso in modulating the differential expression of immune regulatory genes, we first confirmed that Kaiso depletion in MDA-MB-231 cells (sh-Kaiso) altered the expression of the immune regulatory genes THBS1, CD47, and SIRPA at the mRNA and protein levels, both in exosomes and in cells (Figure 3A,B). Significantly increased expression of THBS1, a regulator for anti-angiogenesis and phagocytosis, was observed in sh-Kaiso cells (relative expression = 11.1585, *p* ≤ 0.0039) and exosomes (relative expression = 6.5542, *p* ≤ 0.0001) (Figure 3B). In contrast to THBS1 expression, Kaiso depletion (relative expression = 0.158517, *p* ≤ 0.0045) exhibited decreased expression of the “don’t eat me” signal protein CD47 (cells: relative expression = 0.615979, *p* ≤ 0.0333; Exo: relative expression = 0.291455, *p* ≤ 0.0046) as well as its counter receptor SIRPA (cells: relative expression = 0.0855693, *p* ≤ 0.0212; Exo: relative expression = 0.285693, *p* ≤ 0.0049). The association of these two molecules assists cancer cells in evading immune surveillance in multiple tumor types [64,65,66].

### 3.5. Increased Expression of Kaiso Correlates with Copy Number Alteration in Breast Cancer

To determine the biological significance of Kaiso with the THBS1/CD47/SIRPA signaling axis, we first analyzed two datasets that contained gene expression from commonly utilized breast cancer cell lines. We found that breast cell lines with high Kaiso expression showed a positive correlation with CD47 and SIRPA gene expression, while THBS1 expression correlates negatively with Kaiso level (Supplementary Figure S4). Altogether, BRCA cell line data revealed that high Kaiso/CD47/SIRPA expressed cells expressed low THBS1, and this was further correlated with a similar pattern of copy number alternation.

### 3.6. Kaiso Directly Regulates THBS1 via Modulation of Transcriptional Repression of THBS1

The characteristics of increased metastasis by evading immune surveillance in breast cancer can be depicted by the alternative immune signaling and reduced expression of tumor suppressors, such as THBS1. Previously, our lab showed that Kaiso influences EMT and metastasis through direct binding to methylated regions in the E-cadherin promoter [23]. Similarly, in multiple cancer types, the expression of THBS1 is silenced through hyper-methylation of its promoter to induce immune escape and EMT [67,68]. We performed CHIP assays to determine whether Kaiso binds to methyl-CpG dinucleotides in the promoter region of the THBS1 gene. Several CHIP primers (Table S2) were used to amplify a 239-bp fragment of the THBS1 promoter (Figure 3C). Immunoprecipitation was performed by incubating the chromatin complex with a Kaiso antibody or IgG antibody (negative control). DNA amplification by using a CHIP primer amplifying the CpG region of the THBS1 promoter showed positive binding by both PCR and gel electrophoresis (Figure 3D,E). To further determine if Kaiso binds to the methylated area of the THBS1 promoter, we treated MDA-MB-231 cells with the demethylation agent 5-aza to abrogate this binding; the Kaiso antibody did not enrich the methyl-CpG dinucleotides fragment within the THBS1 promoter (Figure 3D,E). These results indicate that Kaiso binds to the THBS1 promoter region in a methylation-dependent manner.

### 3.7. Kaiso Depletion Attenuates the In Vivo Tumor Growth of Triple-Negative Breast Cancer (TNBC) Cells

**Figure 4:**
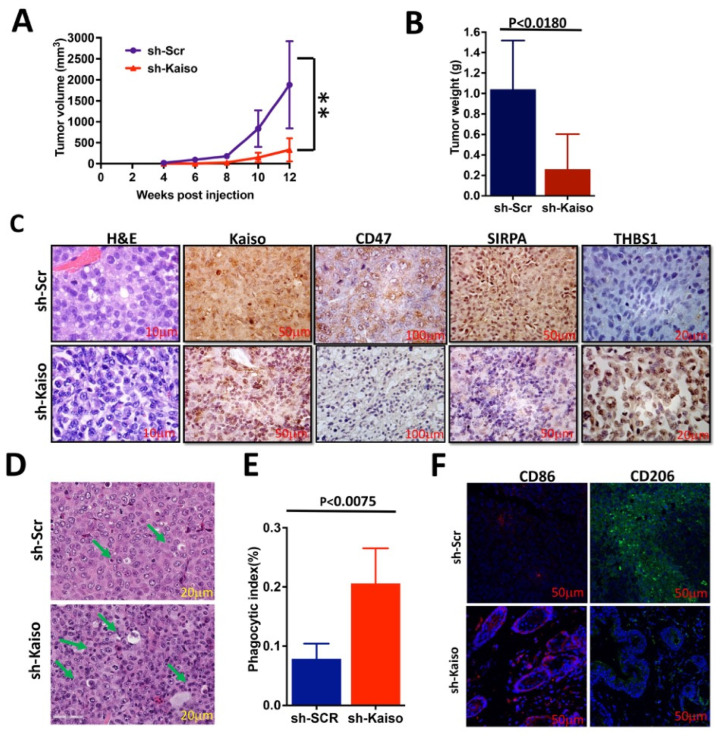
Kaiso depletion attenuates the growth of TNBC cells. (**A**) Kaiso-depleted MDA-MB-231 xenograft tumors (sh-Kaiso) showed delayed tumor onset (~4 weeks) and development compared to control MDA-MB-231 (sh-Scr) xenograft tumors, as demonstrated by time-course analysis of tumor volumes in nude mice. The statistical method used was the F test to compare variances between sh-Scr and sh-Kaiso groups (** statistically significant *p* < 0.01) (**B**) sh-Kaiso cells also developed smaller tumor sizes and reduced tumor weights as compared to sh-Scr cells. The statistical method used was the two-tailed paired *t* test. (**C**) IHC-stained images of xenograft tissues with Kaiso, CD47, and SIRPA antibodies showed a marked decrease in intensity but an increase in THBS1 in Kaiso-depleted (sh-Kaiso) tumor tissues. (**D**,**E**) Hematoxylin and eosin (H&E) staining showed significantly increased phagocytosis (green arrow showed phagocytosis) in sh-Kaiso xenograft tissues as compared to sh-Scr xenograft tissues. The statistical method used was the two-tailed paired *t* test. (**F**) Immunofluorescence images of xenograft tissues showed that sh-Kaiso tissues have a high intensity of CD86 and a low intensity of CD206, an M1 macrophage marker, whereas Kaiso control sh-Scr tissues had a low intensity of CD86 and a high intensity of CD206, an M2 macrophage marker.

The established role of Kaiso in basal-type breast cancer has been associated with poor survival [22,23,24,44]. We hypothesized that Kaiso is involved in immune evasion and thereby in tumor growth by modulating the THBS1/CD47/SIRPA axis. To determine the role of Kaiso on this axis, we performed experiments using sh-Kaiso and sh-Scr cells. There was an 8-week delay in tumors becoming palpable with sh-Kaiso cells when compared to sh-Scr cells, which were palpable after only 4 weeks (Figure 4A). This delay in the onset of tumor development was associated with a smaller tumor size after 12 weeks and lower (*p* < 0.0180) average tumor weights (Figure 4B), as previously reported [44]. IHC analysis for Kaiso, CD47, and SIRPA expression demonstrated that, similar to the in vitro studies, THBS1 expression was increased, and CD47 and SIRPA were decreased in sh-Kaiso tissues (Figure 4C). Interestingly, CD47 was highly expressed in the surface membrane as well as in the cytoplasm, and in the sh-Scr tissues as compared to sh-Kaiso tissues. Cytoplasmic expression of CD47 may be due to the interaction of CD47 with other molecules, especially in the basal subtype. The expression of CD47 in the study is consistent with previous studies where the authors showed the membrane and cytoplasmic expression of CD47 in tissues taken from TNBC and other breast subtypes [69,70]. H&E-stained images from sh-Kaiso tumor tissues also showed significantly increased (*p* < 0.0075) phagocytosis as compared to sh-Scr tumor tissues (Figure 4D,E). To characterize this phenomenon, we performed immunofluorescence staining of tissues for M1 (CD86) and M2 (CD206) markers. We observed low CD86 and high CD206 in sh-Scr tissues (Figure 4F), changes that were reversed in sh-Kaiso tissues with high CD86 and low CD206.

### 3.8. Exosomes Act as Cell-to-Cell Communication Vehicles

**Figure 5:**
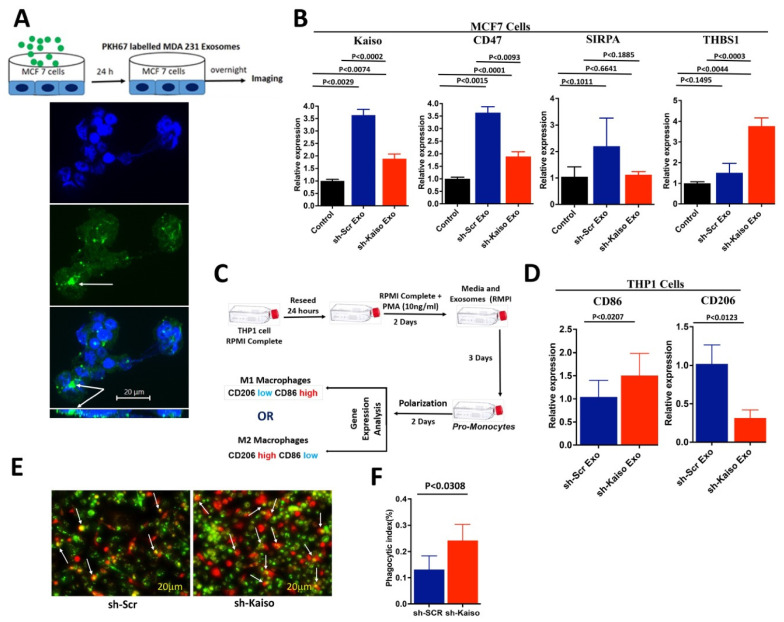
Exosomes act as cell-to-cell communication vehicles influencing MCF7 cells following treatment with exosomes. (**A**) Internalization of exosomes from MDA-MB-231 cells was confirmed by the uptake of exosomes in recipient MCF7 cells by using PKH67 polylinker dye (white arrows highlight exosome in Z-stack image). (**B**) MCF7 cells treated with Kaiso-depleted exosomes showed low expression of Kaiso, CD47, and SIRPA, but high expression of THBS1. The statistical method used was one-way ANOVA. (**C**) Polarization of THP1 cells using exosomes from Kaiso-depleted cells was assessed to demonstrate the influence of exosomes as communication vehicles by observing the expression of M1 and M2 macrophage markers. (**D**) Consistent with sh-Kaiso xenograft tissues (Figure 4F), THP1 cells treated with exosomes from sh-Kaiso cells showed a high expression of CD86 and a low expression of CD206, a marker for M1 macrophages; in contrast, THP1 cells treated with exosomes from sh-Scr cells showed a low expression of CD86 and a high expression of CD206, a marker for M2 macrophages. The statistical method used was the two-tailed paired *t* test. (**E**,**F**) BMDM treated with sh-SCR and sh-Kaiso cells showed higher phagocytosis (white arrow indicates phagocytosis) of sh-kaiso cells as compared to sh-SCR cells. The statistical method used was the two-tailed paired *t* test.

To determine if Kaiso influences exosomes to promote THBS1, CD47, and SIRPA, we extracted exosomes from sh-Kaiso and sh-Scr cells and then used them to treat MCF7 cells (Figure 5). To confirm that exosomes were internalized by recipient MCF-7 cells, both sh-Kaiso and sh-Scr exosomes were labeled with PKH green. MCF7 cells were treated with labeled exosomes for 4 h. Using fluorescence microscopy, we observed that MCF-7 cells internalized exosomes (Figure 5A). Next, we validated the expression of CD47, SIRPA, and THBS1 in exosome treated MCF7 cells. THBS1 expression was increased (*p* < 0.0003) in sh-Kaiso exosome-treated MCF7 cells, whereas expressions of Kaiso (*p* < 0.0002), CD47 (*p* < 0.0093), and SIRPA (*p* < 0.1885) were decreased (Figure 5B), indicating the influence of Kaiso in exosome-mediated immune gene expression. To confirm the functional role of Kaiso/THBS1/CD47/SIRPA on macrophage polarization, we treated THP-1 cells with exosomes from sh-Kaiso and sh-Scr cells (Figure 5C). We found that sh-Kaiso exosome-treated THP1 cells showed a high expression of CD86 (*p* < 0.0207) and a low expression of CD206 (*p* < 0.0123), an M1 macrophage phenotype; in contrast, sh-Scr exosome-treated THP1 cells showed a low expression of CD86 and a high expression of CD206, an M2 macrophage phenotype (Figure 5D). We then co-cultured BMDMs with cancer cells to observe whether Kaiso influences preventing phagocytosis, and we found that a significantly high number of sh-kaiso cells were phagocytosed by BMDMs as compared to sh-SCR cells (Figure 5E,F). These results indicate that Kaiso influences M2 macrophage polarization, which is tumor supportive, compared to M1 polarization, which has anti-tumor activity.

### 3.9. Increased Expression of Kaiso Correlates with Basal Barest Cancer Patients and Reduced Patient Survival

**Figure 6:**
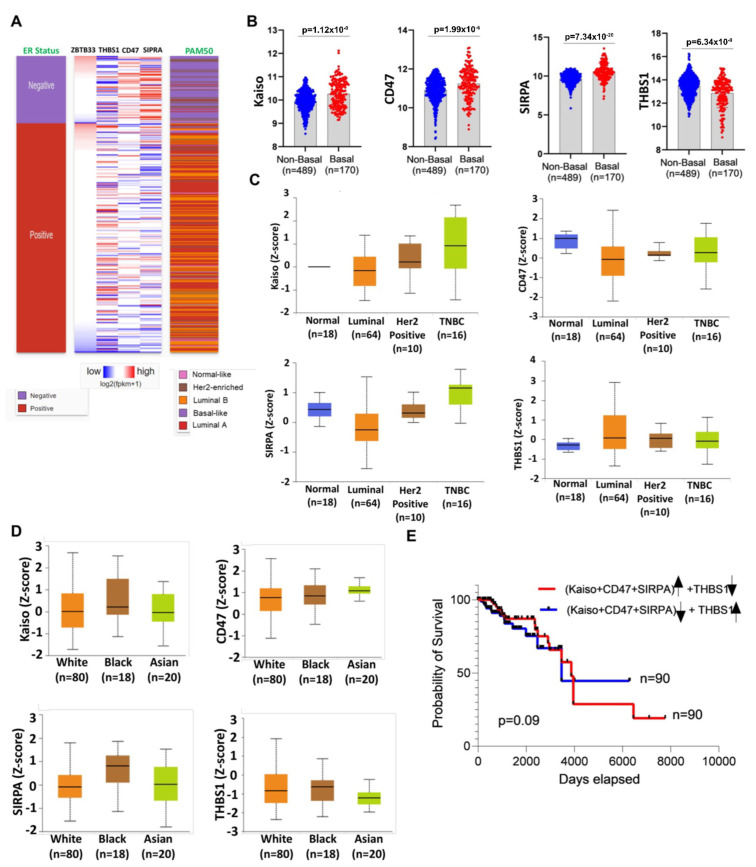
TCGA data validate the increased expression of Kaiso and CD47, with decreased expression of THBS1 correlating with TNBC breast cancer patients and linked with AA race. (**A**) High expression of Kaiso, CD47, SIRPA, and low expression of THBS1 was correlated with Basal-like BRCA and ER-negative patients. (**B**) Kaiso, CD47, and SIRPA expressed highly in Basal like patients as compared to non-basal patients, whereas THBS1 expression was deceased in Basal like patients as compared to non-basal patients. (**C**) The higher level of protein for Kaiso, CD47, and SIRPA and lower level of THBS1 observed in BRCA TNBC samples as compared to other subtypes collected from the PANCAN RPPA dataset. (**D**) The higher level of protein for Kaiso, CD47, and SIRPA in AA patient samples as compared to other races. (**E**) TCGA survival analysis identified that a high level of combined Kaiso, CD47, and SIRPA with a low level of THBS1 (Red line) indicate even poorer patient survival in AA BRCA patients compared to a low level of combined Kaiso, CD47, and SIRPA with a high level of THBS1 (Dark blue line).

To determine the clinical significance of Kaiso with the THBS1/CD47/SIRPA signaling axis in breast cancer patients, we analyzed both the TCGA gene expression and protein dataset. We found that high Kaiso expression is correlated with low expression of THBS1, and high expression of CD47 and SIRPA. This expression profile was more pronounced in patients that have a basal-like subtype and are ER-negative (Figure 6A). We observed consistence expression of this gene signature in all basal-like patients as compared to non-basal patients (Figure 6B). Because we have reported that Kaiso protein expression is significantly overexpressed in African American women, we next sought to determine if our gene signature was consistent with protein expression using the PANCAN RPPA dataset. Interesting Kaiso and SIRPA protein expression was elevated in the TNBC subtype (Figure 6C) and black patients (Figure 6D) compared to CD47 and THBS, which did not show any significant difference across breast cancer subtypes, suggesting these markers could be the important markers for AA patients. We did analyze both the gene/protein signature based on cancer stage; however, we did not observe any significant difference in these markers. Finally, to decipher if this signature affects overall survival, we performed Kaplan–Meier curves in AA patients. Interestingly, we observed a lower survival in AA patients of a high expression of Kaiso, CD47, and SIRPA with a low expression of THBS1 as compared to a low expression of Kaiso, CD47, and SIRPA with a high expression of THBS1 (*p* = 0.09), although these findings were not statistically significant (Figure 6E). Collectively, our findings suggest that Kaiso, CD47, SIPRA, and THBS1, gene signature is related to ER-negative basal-like tumors, which includes TNBC breast cancer patients. Both clinical characteristics have been well-documented to be more frequent in African American breast cancer patients.

## 4. Discussion

Exosomes and their contents contribute to numerous biological functions, including induction of the EMT and modulation of host immunity to escape immune response [59,71,72]. Our group and others have reported that protein expression of the transcriptional repressor Kaiso is elevated in AA breast cancers [22], and it inhibits the expression of tumor suppressor genes that promote EMT-associated metastasis [23,73,74]. As cells undergo the transition from the epithelial to the mesenchymal phenotype, there is a robust network of cell-to-cell communication and subsequent intracellular signaling that allow cancer cells to evade immune surveillance and metastasize. The breast tumors of AA women exhibit a more suppressive immune microenvironment with upregulation of T-cell exhaustion markers. This appears to be linked to Kaiso expression. Using multiplex spatial analysis, we observed that, for AAs, PDL1-positive T-cells and CD68-positive macrophages are in close proximity to high-expressing Kaiso tumors [24]. To explain the racial disparity in breast cancer immune signaling in exosomes, we conducted immune profiling of exosomes extracted from the serum of AA and EA breast cancer patients. We found differential expression of both innate and adaptive gene signatures, with CD47, CCL5, PPBP, and TGFB1 increased in AA exosomes, whereas CD24, IL26, CTSL, and GAGE1 were increased in EA exosomes. Because CD47, the “don’t eat me” signal, is a target for many cancer types, including breast tumors, understanding the molecular events that increase CD47 expression is a clinical need.

In recent years, exosomes have become appreciated as mediators of cell-to-cell communication. To evaluate the influence of exosomes on cell-to-cell communication, we performed immune profiling of MDA-MB-231 cells with depleted Kaiso (sh-Kaiso). As in our patient data, THBS1, a ligand for CD47 [75], is inversely correlated with CD47 expression. We next treated breast cancer MCF7 cells with exosomes from Kaiso high expressing MDA-MB-231 cells and sh-Kaiso cells. Exosomes from Kaiso-depleted cells induced expression of THBS1 and decreased expressions of CD47 and CD47 ligand SIRPA. Because CD47 has diverse functions, the interaction of SIRPA/CD47 is important for the direct regulation of migration and phagocytosis [76], and THBS1 signaling through CD47 modulates the transcriptional regulation of multiple immune response genes and subsequent activation [75,77,78]. For example, ligation of CD47 by THBS1 inhibits the inflammatory response by disrupting the interaction of CD14 and CD47 [79]. Ligation between THBS1 and CD47 also inhibits TLR-dependent transcriptional induction NFKB/AP-1 and promotes type I interferon response [80]. In addition, THBS1 functions as a positive modulator of innate anti-tumor immunity by attracting M1 macrophages to the sites of inflammation or injury and stimulating reactive oxygen species (ROS)-mediated tumor cytotoxicity [75,77]. Because Kaiso appears to be associated with suppressive cytokines, we used sh-Kaiso exosomes or exosomes from control MDA-MB-231 cells to polarize human THP1 macrophages. Exosomes or conditioned media from MDA-MB-231 cells polarize THP1 cells towards the M2 phenotype with the upregulation of CD206 and decreased expression of CD86. However, exosomes from sh-Kaiso cells induced more M1 polarization, whereas THP1 cells showed high expression of the M1 marker CD86 and low expression of the M2 marker CD206. We further confirmed this observation in vivo. sh-Kaiso cells exhibit delayed tumor growth compared to controls [22,23]. Moreover, when we examined the protein expressions of our panel of markers, sh-Kaiso tumors demonstrated low to absent CD47 and CD206 and high expression of THBS1 and CD86. These findings suggest that high Kaiso expression prevents the polarization of macrophages towards the anti-tumor M1 phenotype, both in vitro and in vivo. In further support of this relationship, increased expression of THBS1 and decreased expression of CD47 and SIRPA induce recruitment of M1 macrophages, causing an increase in phagocytosis [81,82,83,84]. The SIRPA–CD47 pathway, known as a phagocytosis checkpoint in macrophages and other immune cells that are involved in avoiding phagocytosis, is considered a therapeutic target [85,86].

Based on previous reports from our lab and others, Kaiso is associated with EMT reprogramming in high-grade breast and prostate tumors [23,44,73,74,87], Kaiso may also mediate immune modulation through the downregulation of THBS1 directly and upregulation of CD47 indirectly. Our findings suggest that Kaiso influences the THBS1/CD47-SIRPA signaling cascade, which is involved in immune surveillance [63,64,65,66,81,88].

Although a limitation of this study is that our in vivo findings were in immunocompromised mice, preventing us from determining the influence of Kaiso on immune cell populations such as T-cells, we were able to examine the Kaiso/CD47/SIRPA/THBS1 relationship for breast cancer patients in two independent datasets, BRCA cell lines, and TCGA [63,64,65,66,81,88]. Additionally, given that the AA and EA patient exosomes did not have a comparable control group, it is possible that the gene expression changes between racial groups observed from serum-derived exosomes may not be fully reflective of all the changes unique to women with breast cancer. We previously reported that Kaiso protein expression is significantly increased in AA and native African patients compared to EA patients [22,24]. Our current analysis of TCGA data and PANCAN_RPPA data and from breast cancer patients show that elevated expression of Kaiso is associated with increased CD47 and SIRPA and decreased THPS1 in ER-negative basal-like tumors. Not surprisingly, because a significant amount of TNBC patients have basal-like phenotype, we also observed this expression in TNBC patients. Similar to our previous IHC results for Kaiso, we observed that Kaiso and now SIRPA protein levels increased in AA breast cancer patients. We did observe that high expression of Kaiso, CD47, SIRPA, and low expression of THBS1 correlates with shorter survival in AA patients, although this was not significant. It is possible this trend was not statistically significant due to the low number of AA patients within the TCGA dataset. Overall, our in vitro, in vivo, and patient data is consistent in that Kaiso plays a regulatory role in THBS1, CD47, and SIRPA, which are each well documented for cancer to evade immune surveillance.

## 5. Conclusions

**Figure 7:**
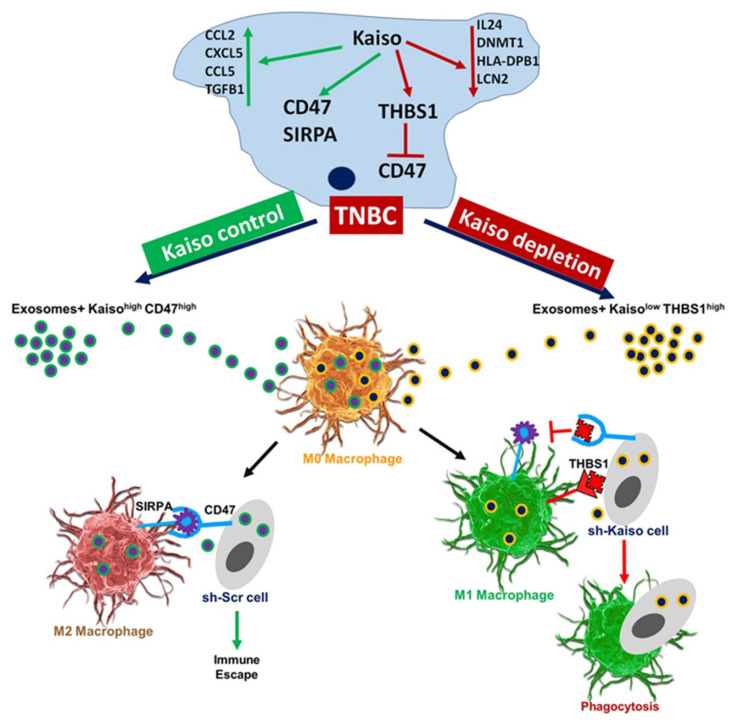
Summary.

In conclusion, our results demonstrate that the downregulation of THBS1 is, in part, regulated by direct Kaiso binding. Depleted expression of Kaiso in breast cancer cells modulates the immune signaling molecules within the cargo of exosomes, resulting in increased THBS1 expression and decreased CD47 and SIRPA expression, as well as in decreased phagocytosis by macrophages (Figure 7). Because Kaiso protein is overexpressed in breast tumors, especially those of AAs, our study provides evidence supporting the biological role of Kaiso in immune signaling. It also indicates that AA breast cancer patients could benefit from targeted CD47 antibody therapies that are currently in clinical trials

Model of the role of Kaiso in immune signaling in breast cancer exosomes. Kaiso depletion results in increased expression of THBS1, which in turn inhibits CD47. Exosomes from Kaiso-depleted cells inhibit CD47 by binding with THBS1 and promoting M1 type macrophage polarization, which makes the cells prone to immune surveillance and increased phagocytosis. Kaiso depletion results in increased expression of THBS1, which in turn inhibits CD47. In contrast, exosomes from cells with high Kaiso expression show high CD47 and SIRPA expression. In Kaiso control cells, CD47 binds with SIRPA and promotes M2-type macrophage polarization, which allows the cells to avoid immune surveillance and to show decreased phagocytosis and immune escape.

## Data Availability

The data presented in this study are available on request from the corresponding author.

## Supplementary Materials

The following are available online at https://www.mdpi.com/article/10.3390/cancers15082282/s1: Figure S1: Characterization of exosomes. Figure S2: Differential expression of chemokines, cytokines, and interleukins function genes in AA as compared to EA breast cancer patient exosomes. Figure S3: Differential expression of immune genes from sh-Kaiso and sh-Scr exosomes. Figure S4. Increased expression of Kaiso, CD47 and SIRPA corelated with decreased expression of THBS1. Table S1: Patient characteristics of collected blood samples. Table S2: List of primers used for CHIP assay. Table S3: List of primers used for PCR assay. File S1: The uncropped blots.
