# Supplementary material for: Immune Profile of Exosomes in African American Breast Cancer Patients Is Mediated by Kaiso/THBS1/CD47 Signaling
Source: Cancers (Basel). 2023 Apr 13;15(8):2282. doi: 10.3390/cancers15082282 (PMC10136634; doi:10.3390/cancers15082282)
Supplement: Supplementary file 1 [file cancers-15-02282-s001.zip › cancers-2136835-supplementary.pdf]

Cancers **2022,**

Supplementary materials:

Immune Profile of Exosomes in African American Breast Cancer Patients is mediated by Kaiso/THBS1/CD47 signaling

Md Shakir Uddin Ahmed[3,4], Brittany D. Lord[5], Benjamin Adu Addai[6], Sandeep S. Singhal[7], Kevin Gardner[8], Ahmad Bin Salam[3], Anghesom Ghebremedhin[3], Jason White[3], Iqbal Mahmud[9], Rachel Martini[10], Deepa Bedi[3], Huixian Lin[3], Jacqueline D. Jones[11], Balasubramanyanam Karanam[3], Windy Dean-Colomb[3], William Grizzle[12], Honghe Wang[3], Melissa Davis[10] and Clayton C Yates[1,2,3*]

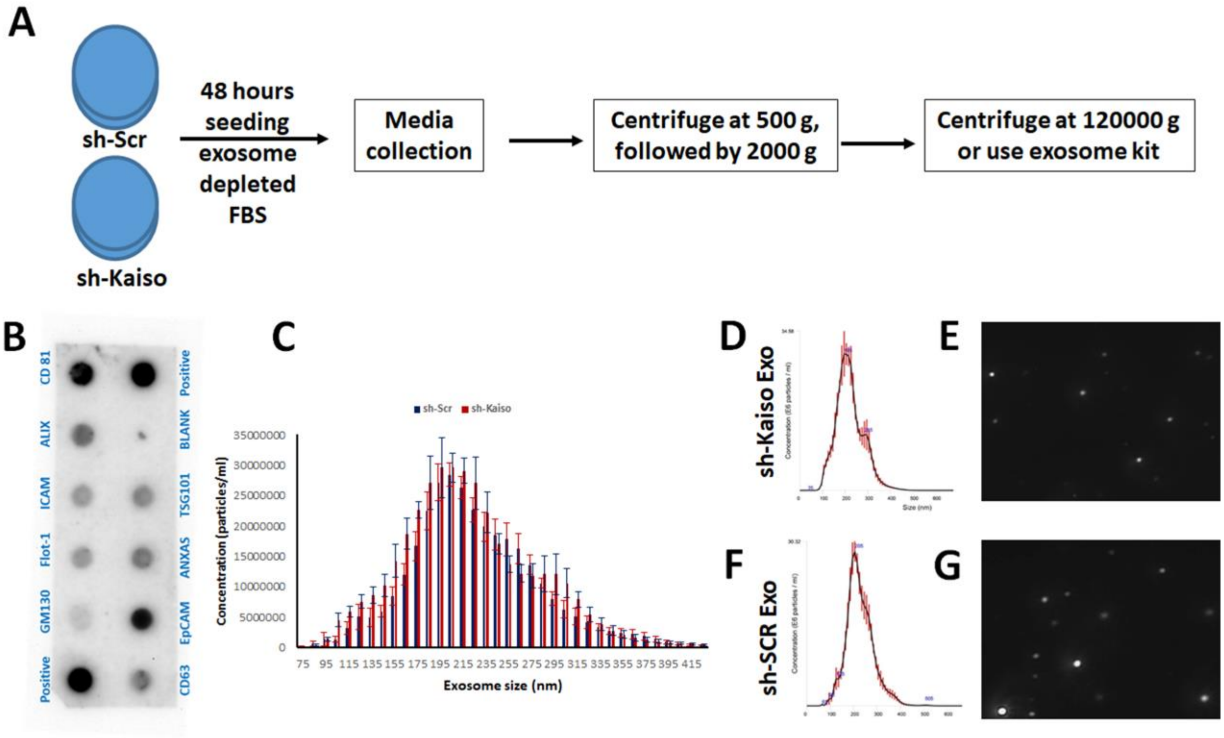

Supplementary Figure S1: Characterization of Exosomes. (A) Extraction protocol for extracting exosomes from cell culture or patient serum using ultracentrifugation or using commercially available exosome extraction kit. (B) Exosome antibody array analysis of exosomes from MDA-MB-231 cells demonstrated that markers for exosomes were present in exosomes. (C)

Nanosight analysis of size distribution of exosomes from sh-Scr and sh-Kaiso cells, showing the average size of the extracted exosomes were ~195-205 nm in diameter. (D, E, F & G) Nanosight analysis showed average size distribution with exosomes images.

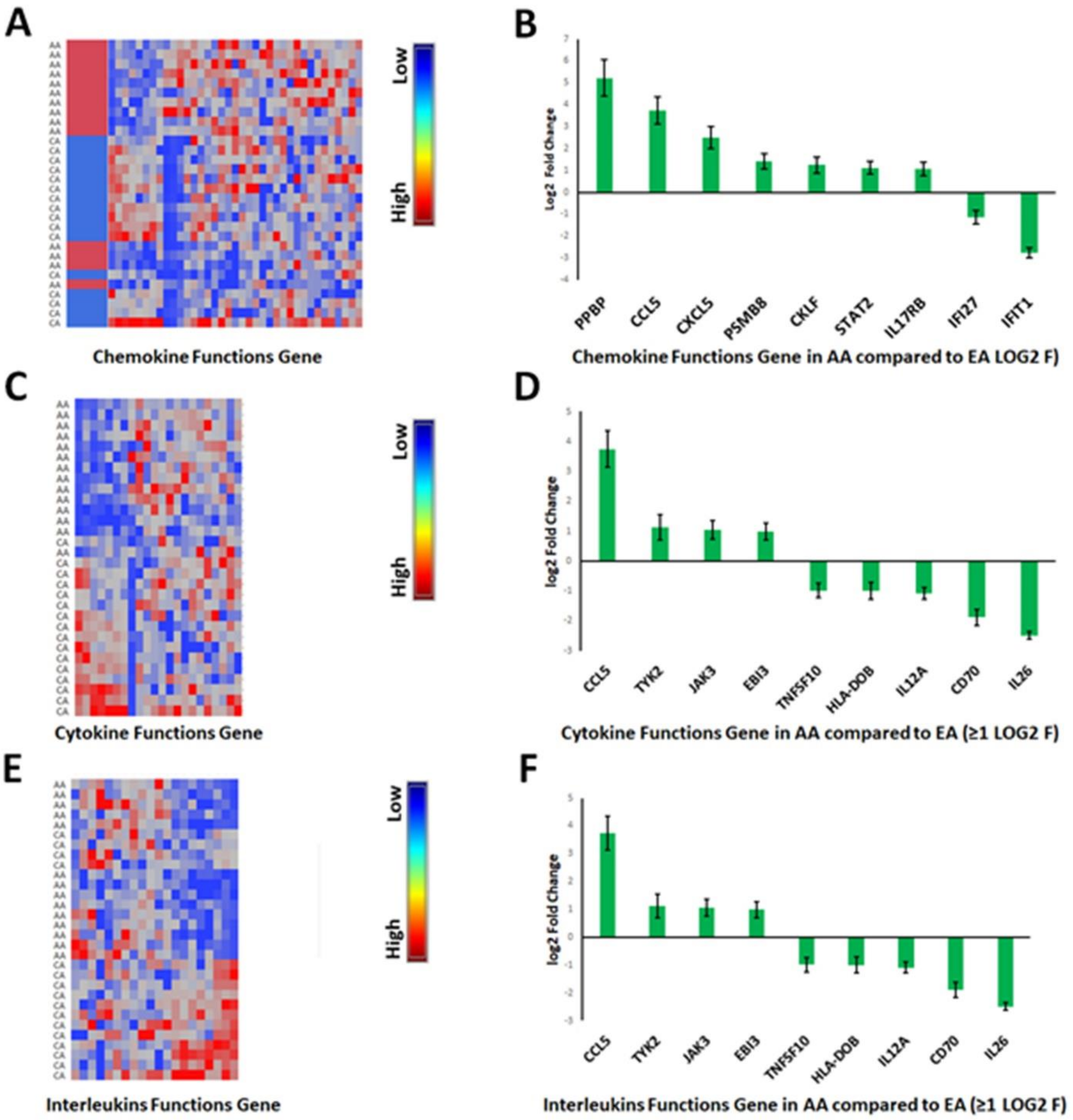

Supplementary Figure S2: Differential expression of chemokines, cytokines and interleukins function genes in AA as compared to EA breast cancer patient exosomes. Nanostring PanCancer Immune gene analysis which includes 730 immune function genes, showed that the immune function genes are expressed differentially among AA and EA breast cancer exosomes. **(**A) Among the chemokine function genes like PPBP, CCL5, CXCL5, PSMB8 are expressed highly in AA exosomes whereas a few chemokine genes like IFI127 AND IFIT1has higher expression in EA breast cancer exosomes. (B & C) As consistent with chemokine function genes, Cytokine and Interleukin function genes are also expressed differentially among AA and EA exosomes.

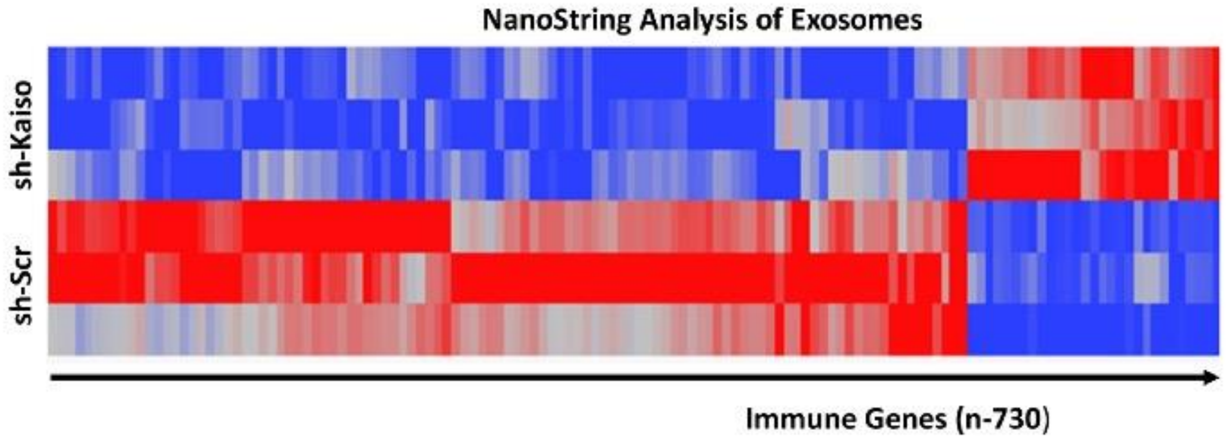

Supplementary Figure S3: Differential expression of immune genes from sh-Kaiso and sh-Scr exosomes. Hierarchical clustering of differentially expressed genes from PanCancer Nanostring Immune Profiling showed that there was fewer immune gene expressed in sh-Kaiso exosomes as compared to sh-Scr exosomes.

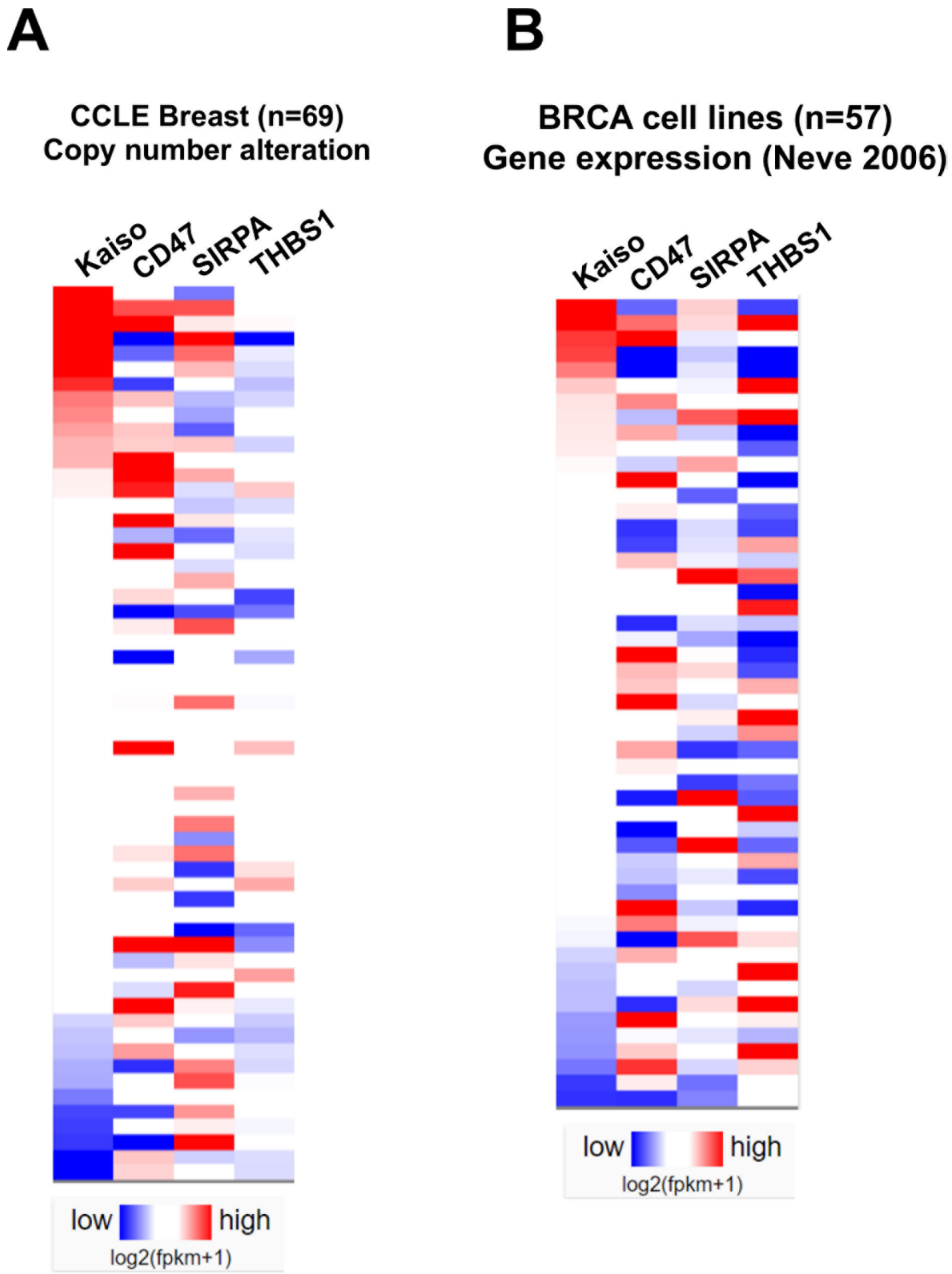

Figure S4. Increased expression of Kaiso, CD47 and SIRPA corelated with decreased expression of THBS1. CCLE and BRCA cell lines gene expression analysis shows that high expression of Kaiso showed positive correlation with high expression of CD47, SIRPA and correlated with low expression of THBS1 in multiple BRCA studies (A & B).

Supplementary Table S1: Patient characteristics of breast cancer patients

Patient Id	Age	Ethnicity	Molecular Subtype	Stage
EEG4	48	African American	Lum A	IV
EEG5	73	African American	Lum A	IIA
EEG8	57	African American	Lum A	IIIA
EEG13	57	African American	HER2+	IA
EEG16	62	African American	Lum B	IIB
EEG19	64	African American	NA	NA
EEG23	74	African American	NA	NA
EEG30	69	African American	Lum A	IIC
EEG80	59	African American	Lum A/B	0, pTisN0M0
EEG108	61	African American	Lum A	IC
EEG118	55	African American	Lum A	IV
EEG125	62	African American	Lum A	II
EEG128	79	African American	NA	NA
EEG135	90	African American	NA	NA
WD-40544	39	European American	TNBC	NA
WD-40505	54	European American	LUMA	Stage IA
WD-40528	31	European American	TNBC	NA
WD-40567	56	European American	LUMA	Stage IA
WD-40510	53	European American	LUMA	Stage IIIA
WD-40552	67	European American	TNBC	Stage IA
WD-40507	60	European American	LUMB	Stage IA

WD-40574	73	European American	LUMB	Stage IA
WD-40526	66	European American	LUMA	Stage IA
WD-40516	74	European American	LUMB	Stage IA
WD-40572	46	European American	LUMB	Stage IIA
WD-40534	66	European American	NA	Stage IA
WD-40500	61	European American	HER2	Stage IA
WD-40576	89	European American	HER2	Stage IIIB
WD-40536	32	European American	HER2	Stage IA
WD-40522	46	European American	NA	NA

Supplementary Table S2: List of primers used for CHIP assay

Target	Product Size (BP)	Annealing Temperature (°C)	Sequence (5'-3')
THBS1-ME-P1-F	239	55.09	CCC CCT TCA CTT TCT AGC TG
THBS1-ME-P1-R		58.40	CCG GAG TAG AGG TTG CTC CT
THBS1-ME-P2-F	231	56.13	AGT ATC CAC CTC TCG CCA TC
THBS1-ME-P2-R		57.06	GGC TTG GGA GCA CTA GAA CTT
CD47-ME-P2-F	237	55.91	GCA GTC ACA AAC CAA GCT CA
CD47-ME-P2-R		58.90	GCT CGT CTA CTC GCT CTG CT
CD47-ME-P4-F	204	55.23	GGT GGG AGT GAA AGC AAA GA
CD47-ME-P4-R		57.45	CTT CCA GGT CAC GTC CTG TC
SIRPA-ME-P1-F	189	55.39	CGG GGT CTT GAA CTT GTG TT
SIRPA-ME-P1-R		55.12	CAA GCC CTG AGG AAG ATG AG
SIRPA-ME-P2-F	179	55.64	GGG AGG GTT AAA AGG CAG AC
SIRPA-ME-P2-R		57.33	GAG GAC GAT GTA GCC AGC TC
TGFB1-ME-P1-F		54.00	GCA AAG ACT TTT CCC CAG AC

TGFB1-ME-P1-R	161	55.99	AGA AGG TGG GTG GTC TTG AA
TGFB1-ME-P4-F	160	54.81	CAA AGA CTT TTC CCC AGA CCT
TGFB1-ME-P4-R		55.99	AGA AGG TGG GTG GTC TTG AA

Supplementary Table S3: List of primers used for PCR assay

Target	Annealing Temperature (°C)	Sequence (5'-3')
Kaiso-P1-F	54.79774531	GAT TCT GCC CAC AAA GGA GA
Kaiso-P1-R	56.23129294	AAC AGG GCC TGG GTT AGA TT
CD47-P2-F	54.52741302	CCT GCA GCA CTT TTT CCT TC
CD47-P2-R	52.97876724	CAA GAG CGA ACC CCA AAT AA
TGFB1-P1-F	56.40585983	GGG ACT ATC CAC CTG CAA GA
TGFB1-P1-R	56.97499128	CCT CCT TGG CGT AGT AGT CG
THBS1-P1-F	54.58566774	TTG TCT TTG GAA CCA CAC CA
THBS1-P1-R	56.54201379	CTG GAC AGC TCA TCA CAG GA
SIRPA-P1-F	51.47858578	CCA TGG AAA AGC TGG AAA AA
SIRPA-P1-R	54.01923458	CAA GGA AGC AGA GGG TTT TG
β-Actin-F	56.71881079	TGG ACT TCG AGC AAG AGA TGG
β-Actin-R	56.57054287	ATC TCC TTC TGC ATC CTG TCG
