# Supplementary figures and images for: Immune Profile of Exosomes in African American Breast Cancer Patients Is Mediated by Kaiso/THBS1/CD47 Signaling
Source: Cancers (Basel). 2023 Apr 13;15(8):2282. doi: 10.3390/cancers15082282 (PMC10136634; doi:10.3390/cancers15082282)

# Uncropped blots for Figure 3B

**KAISO**

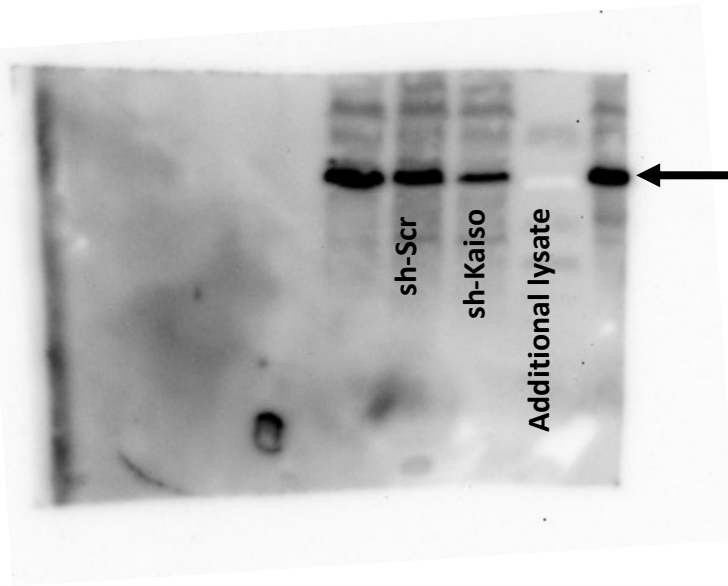

**CD47**

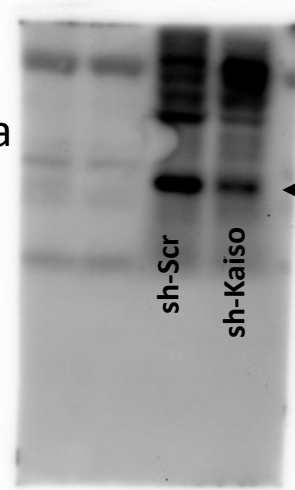

**SIRPA**

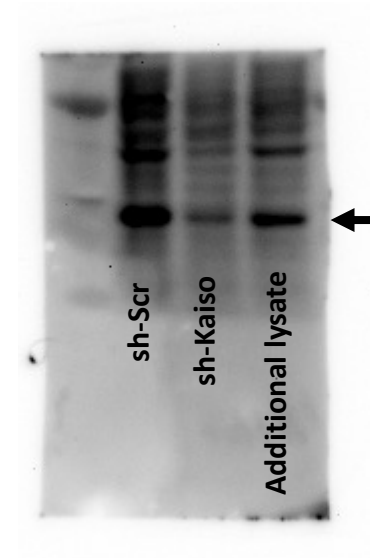

**THBS1**

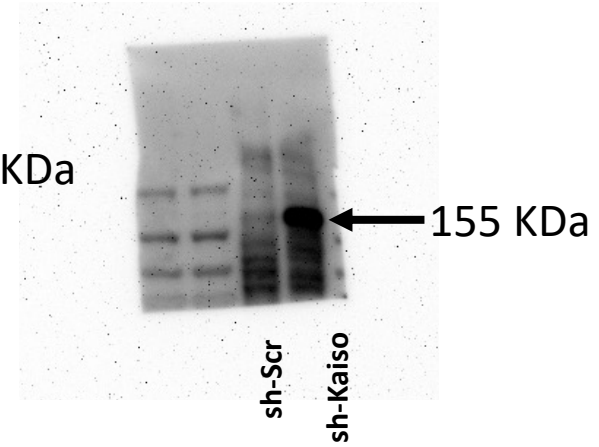

**B-actin**

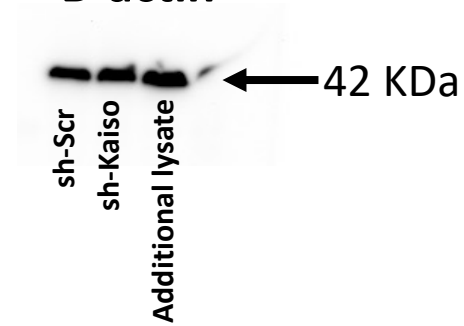

Uncropped blots for figure 3E

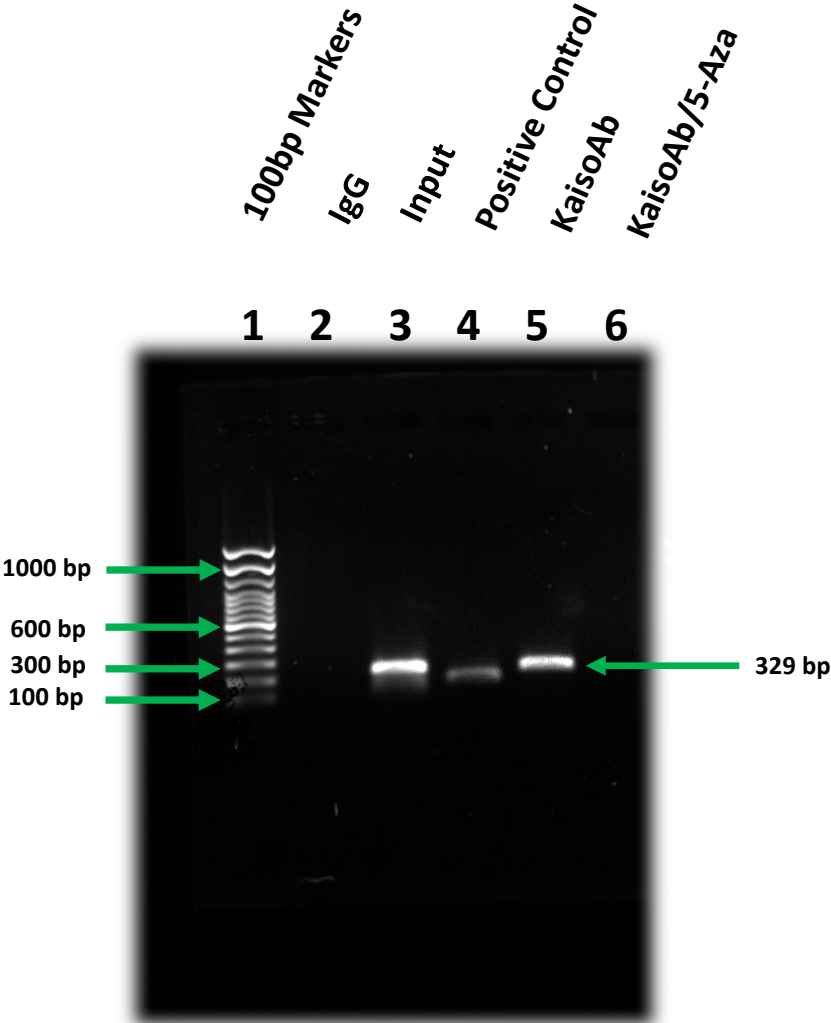

Supplement: Supplementary file 1 [file cancers-15-02282-s001.zip › cancers-2136835-file S1.pdf]
